# Serum and Placental Trace Element Levels, Major Uterine Pathogens, and Reproductive Performance in Simmental Cows During the Early Postpartum Period

**DOI:** 10.1007/s12011-025-04951-0

**Published:** 2026-01-09

**Authors:** Vefa Tohumcu, Damla Tugce Okur, Mehmet Cengiz, Armagan Hayirli, Seyda Cengiz, Cihan Oz, Mehmet Cemal Adiguzel

**Affiliations:** 1 Department of Obstetrics and Gynecology, Faculty of Veterinary Medicine, Atatürk University, Erzurum, 25240 Türkiye; 2 Deparment of Obstetrics and Gynecology, Faculty of Veterinary Medicine, Muğla Sıtkı Koçman University, Muğla, 48000 Türkiye; 3 Department of Animal Nutrition and Nutritional Disorders, Faculty of Veterinary Medicine, Atatürk University, Erzurum, 25240 Turkey; 4 Department of Microbiology, Faculty of Veterinary Medicine, Muğla Sıtkı Koçman University, Muğla, 48000 Türkiye; 5 Department of Microbiology, Faculty of Veterinary Medicine, Atatürk University, Erzurum, 25240 Türkiye

**Keywords:** Cows, Major uterine pathogens, Postpartum period, Reproductive performance, Trace element

## Abstract

This study investigated the associations among serum and placental trace element concentrations, major uterine pathogens isolated from lochia, and reproductive performance during the early postpartum period in cows. Placental samples collected immediately after parturition and blood and lochia samples obtained on days 0, 2, 4, 6, 8, and 10 postpartum from 36 Simmental cows. The primary uterine pathogens identified were Trueperella pyogenes, Escherichia coli, Fusobacterium necrophorum, and Prevotella melaninogenica. Cows with pathogen isolation on consecutive sampling days were classified as infected (IC), whereas cows with no pathogen isolation were classified as non-infected (NIC). Serum Mn, Se, Fe, and Cu concentrations were higher in NIC (*P* < 0.05, *P* < 0.01) than those in IC. However, serum Zn level was higher in IC than NIC. No significant group differences were detected in placental element contents. These findings indicate that alterations in trace element status may influence uterine defense mechanisms and subsequently shape postpartum reproductive performance. Regular monitoring of trace element profiles could therefore support the early detection of imbalances and enable timely interventions to reduce uterine infection risk. Future integrative studies evaluating mineral dynamics across serum, placenta, and lochia alongside microbial colonization may improve understanding of postpartum uterine infections and guide herd-level reproductive management strategies to enhance reproductive performance.

## Introduction

The postpartum period is a critical phase influencing reproductive performance in dairy cows, encompassing uterine involution, histological and bacteriological clearance, re-epithelialization, and the resumption of ovarian cyclicity. In most cows, these processes are completed within approximately 45-60 days after calving, thereby restoring physiological readiness for subsequent conception [1]. During uterine involution, necrosis and detachment of caruncular tissues occur, and the resulting debris is expelled through the vagina as lochia, a viscous, slightly malodorous discharge. Lochia typically persists until about day 20 postpartum and consists of blood, uterine exudate, remnants of fetal membranes, and sloughed endometrial tissue. It provides valuable insight into the progress and completeness of uterine involution and constitutes a favorable medium for bacterial growth [2–5]. Owing to cervical dilation during parturition, bacterial contamination of the uterus is common in the early postpartum period. However, not all cows progress from contamination to infection. The progression to infection depends on several factors, including microbial load, pathogen virulence, the uterine environment, and host immune competence. A high microbial burden, combined with impaired immune defenses, predisposes cows to uterine infection [1, 6, 7].

The pathogens most frequently associated with postpartum metritis include *Escherichia coli*,* Trueperella pyogenes*,* Fusobacterium necrophorum*,* and Prevotella melaninogenica* [8–10]. These organisms are consistently reported as the major etiological agents of postpartum uterine infections and are known to cause abnormal vaginal discharge, disrupt the hypothalamic -pituitary- ovarian axis, suppress estradiol secretion and follicular development, and ultimately reduce conception rates [11].

Phagocytic cells play a pivotal role in the microbial clearance of the postpartum uterus. In addition to cytokines, chemokines, and antioxidants that regulate their activity, certain trace elements also influence this process. Trace elements contribute to uterine health by mitigating oxidative stress, enhancing immune function, and exerting antibacterial effects. In particular, copper (Cu), zinc (Zn), iron (Fe), selenium (Se), and manganese (Mn) are essential for supporting cellular immunity and inhibiting bacterial proliferation [12–14]. Moreover, because the placenta is in direct contact with the uterine lumen and lochia, its element composition may not fully mirror systemic status but could influence the local microenvironment and bacterial colonization dynamics. This relationship has been scarcely addressed in the literature; however, it has been suggested that the decomposing placental membrane together with lochia provides an optimal intrauterine environment for microbial growth [15]. Therefore, the aim of this study was to determine the concentrations of trace elements in serum and placental samples collected during the early postpartum period in cows, to evaluate the associations between these elements and the presence of major uterine pathogens in lochia, and to assess the potential implications of these parameters for postpartum uterine health and reproductive performance.

## Materials and Methods

### Animals and Experimental Design

This study was conducted at the Dairy Cattle Unit of Atatürk University Food and Livestock Application and Research Center. All procedures were approved by the Local Ethics Committee on Animal Experiments of Atatürk University (Approval No: 2020/127). A total of 36 Simmental cows with parity ranging from 2 to 5 were included. Simmental, a dual-purpose breed widely used in Turkey, was selected because it represents typical regional management conditions and has high adaptability.

No cows received antibiotic or anti-inflammatory treatment before or after calving. All animals were maintained under identical management conditions and fed ad libitum from the prepartum period until day 10 postpartum. All cows were fed twice daily with the same total mixed ration (TMR), formulated according to NRC recommendations and consisting of corn silage, alfalfa hay, barley, and a commercial dairy concentrate enriched with mineral-vitamin premix. The same ration was provided to all animals throughout the study period. Only clinically healthy cows that calved at full term (283 ± 3 days) and had no metabolic or hoof disorders were included.

All calvings were continuously monitored, and any complications were recorded. Placentas were naturally expelled within six hours of parturition and immediately collected under appropriate conditions. Blood and lochia samples were collected on days 0, 2, 4, 6, 8, and 10 postpartum (D0-D10). Cows from which at least one of the major uterine pathogens (*Escherichia coli*,* Trueperella pyogenes*,* Fusobacterium necrophorum*,* Prevotella melaninogenica*) was isolated on consecutive days were classified as infected (IC, *n* = 28), whereas cows with no isolation of these pathogens were classified as non-infected (NIC, *n* = 8).

## Sample Collection

At calving, calf birth weight and expelled placental weight were recorded using a precision scale. Placental samples were collected on day 0 from at least three distinct regions (caruncular and intercaruncular areas) and stored at -80 °C. Lochia samples were collected between D0 and D10 from the *vaginal fornix* and *portio vaginalis* using double-guarded swabs under aseptic conditions. Prior to sampling, the vulva and perineal area were disinfected with 70% ethanol. Swabs were placed into sterile tubes containing physiological saline and transported to the laboratory under cold-chain conditions.

Blood samples were collected into 10 mL vacuum tubes from the median caudal vein (*Vena caudalis mediana*). All blood samples were obtained in the morning (between 08:00 and 09:00), prior to milking and before the delivery of the total mixed ration.

## Laboratory Preparation

Samples were delivered to the laboratory within two hours under cold chain conditions. Blood samples were centrifuged at 500 g for 10 min, and sera were stored at − 80 °C until element analysis. Placental samples were homogenized under sterile conditions, and 1 mL homogenate was prepared for element analysis.

## Element Analysis

All standard solutions were prepared using ultrapure water obtained from a Milli-Q purification system (Millipore Corp., Bedford, MA, USA). Stock standard solutions of Mn, Fe, Cu, Zn, and Se (1000 mg/L) were prepared using ultrapure grade water and CertiPUR^®^ multi-element standards (Merck, Poole, UK). For analytical validation, a certified reference material for trace elements in animal serum (NIST SRM 1598a; National Institute of Standards and Technology, Gaithersburg, MD, USA) was analyzed concurrently. All polypropylene tubes used for sample and standard preparation were pre-cleaned by soaking in 10% HNO₃ for 24 h, rinsed thoroughly with deionized water, and air-dried in a laminar flow hood before use.

Elemental determinations were performed using an inductively coupled plasma mass spectrometer (ICP-MS; Agilent 7800x, Agilent Technologies, Tokyo, Japan) equipped with a collision/reaction cell for interference reduction. The sample introduction system consisted of an autosampler, a Scott double-pass spray chamber, a glass concentric MicroMist nebulizer (Glass Expansion, West Melbourne, Australia), a quartz torch, and nickel sampling and skimmer cones. Instrument control and data acquisition were performed using MassHunter Workstation Software (version A.8.01.01, Agilent Technologies, Tokyo, Japan).

Calibration curves were constructed immediately prior to analysis using freshly prepared multi-element standard solutions. Linear regression analyses yielded correlation coefficients (R²) greater than 0.999. Analytical precision was verified by triplicate measurements of each sample, with relative standard deviation (RSD) values below 5%. Procedural blanks and quality control samples were analyzed in parallel. Accuracy was further confirmed by recovery rates ranging between 95 and 105% based on the reference material.

For sample digestion, 1 mL of each serum and placental sample was treated with 2 mL of 30% HNO₃ (Merck, Darmstadt, Germany) and 3 mL of 70% H₂O₂ (Merck, Darmstadt, Germany) using a closed-vessel microwave digestion system (Ethos Up, Milestone^®^). The digestion program consisted of three sequential steps: 145 °C for 5 min, 180 °C for 10 min, and 100 °C for 10 min. The digested solutions were then diluted to a final volume of 25 mL with ultrapure water. Trace element concentrations (Mn, Fe, Cu, Zn, and Se) were quantified by ICP-MS and expressed as mg/dL for serum and mg/g for placental tissue.

For each element, the limits of detection (LOD) and limits of quantification (LOQ) were calculated according to IUPAC recommendations, using three times (LOD = 3σ) and ten times (LOQ = 10σ) the standard deviation of procedural blanks. The calculated LOD values were 0.05 µg/L for Mn, 0.10 µg/L for Fe, 0.08 µg/L for Cu, 0.05 µg/L for Zn, and 0.02 µg/L for Se. The corresponding LOQ values were 0.15 µg/L for Mn, 0.30 µg/L for Fe, 0.25 µg/L for Cu, 0.15 µg/L for Zn, and 0.06 µg/L for Se. All measured concentrations in both serum and placental samples were above the LOQ for the respective elements.

## Bacteriological and Molecular Identification

Bacteriological analyses were conducted at the Department of Microbiology, Faculty of Veterinary Medicine, Atatürk University. Lochia samples were cultured on Columbia agar with 5% sheep blood and MacConkey agar, and incubated under aerobic, anaerobic, and microaerophilic conditions at 37 °C for 2-5 days. Colonies were identified based on morphology, hemolysis patterns, pigmentation, Gram staining, and standard biochemical tests. Pure cultures of *E. coli*,* T. pyogenes*,* P. melaninogenica*, and *F. necrophorum* were stored in tryptic soy broth with 15% glycerol at -80 °C [10].

For molecular confirmation, polymerase chain reaction (PCR) was performed. Genomic DNA was extracted using the boiling method. Colonies were resuspended in 1 mL of TE buffer (pH 8.0; 10 mM Tris-HCl, 1 mM EDTA) containing 10 µL of proteinase K (5 mg/mL) [16]. Each 25 µL PCR reaction contained 2 µL template DNA, 2.5 pmol/µL of each primer, 2.5 µL of 10X PCR buffer, 1.5 mM MgCl₂, 2.5 mM each dNTP, and 0.2 µL Taq DNA polymerase. PCR cycling conditions included an initial denaturation at 94 °C for 2 min; 35 cycles of denaturation at 94 °C for 30 s, annealing at 57-63 °C for 30 s, and extension at 72 °C for 40 s; followed by a final extension at 72 °C for 5 min. PCR products were visualized on 1% agarose gel electrophoresis at 200 V for 30 min [17–21]. Only the four major uterine pathogens were considered. Other bacteria occasionally isolated (e.g., *Acinetobacter spp.*,* Staphylococcus aureus*,* Enterococcus faecalis*,* Mannheimia haemolytica*,* Clostridium spp.*,* Corynebacterium spp.*,* Enterobacter aerogenes*) were excluded as potential contaminants or secondary pathogens.

### Reproductive Parameters

Reproductive performance was evaluated using multiple parameters. Recorded data included parity, calf birth weight, placental weight, and the number of inseminations required to achieve conception. Artificial inseminations were performed by certified technicians using standard estrus detection and synchronization protocols. Pregnancy diagnosis was carried out by transrectal ultrasonography at 30–35 days post-insemination.

### Statistical Analysis

A priori power analysis was conducted using variance estimates derived from our pilot dataset (*n* = 8) collected from cows within the same herd and under identical sampling conditions. Based on the pilot standard deviation and an anticipated medium effect size for differences among uterine health groups, a minimum of 30 animals was required to achieve 80% power at α = 0.05. To compensate for potential exclusions, we enrolled 36 cows, which satisfied the calculated sample size requirement.

Descriptive statistics (mean ± standard deviation) for cow-related variables (parity, insemination number, calf birth weight, placental weight) were obtained using SAS software (Cary, NC, USA). Data normality was assessed using the Shapiro-Wilk test, and homogeneity of variances was tested by Levene’s test. Comparisons between NIC and IC groups at parturition (D0) were performed using the PROC GLM procedure. Pearson’s correlation coefficients were calculated to examine associations between serum and placental element concentrations. Changes in serum element concentrations over time (D2 - D10) were analyzed using repeated measures ANOVA. Mauchly’s test was used to assess the sphericity assumption; when violated, the Greenhouse-Geisser correction was applied. Post-hoc pairwise comparisons were adjusted using the Bonferroni correction. Statistical significance was set at *P* < 0.05. Results are expressed as mean ± SD (Table 1).

## Results

### Descriptive Statistics

The average parity of the 36 Simmental cows included in the study was 2.61 ± 1.48, and the mean number of inseminations per conception was 1.67 ± 1.29. The mean placental weight was 6.35 ± 1.19 kg, and the average birth weight of the calves was 43.8 ± 6.3 kg (Table 2).

**Table 1:** PCR primers and conditions utilized for the molecular verification of isolates

Bacterium target gene	Primer sequence (5’ − 3’)		Annealing temperature (°C)	Band length (bp)	References
*Trueperella* *pyogenes*	plo	F - GGCCCGAATGTCACCGC R - AACTCCGCCTCTAGCGC	55	270	[18]
*Escherichia* *coli*	fimH	F -TGCAGAACGGATAAGCCGTGG R -GCAGTCACCTGCCCTCCGGTA	63	508	[19]
*Fusobacterium necrophorum*	IktA	F -AATCGGAGTAGTAGGTTCTG R -CTTTGGTAACTGCCACTGC	60	401	[21]
*Prevotella melaninogenica*	phyA	F-CGTCATGAAGGAGATTGG R- ATAGAACCGTCAACGCTC	54	389	[20]

**Table 2:** Descriptive statistics

Parameter	Mean ± SD	Rank
Parity	2.61 ± 1.48	1–5
Number of Inseminations	1.67 ± 1.29	0–5
Calf weight	43.8 ± 6.3	31.0–58.0
Placenta weight	6.35 ± 1.19	4.00–9. 50

### Reproductive Performance

There were no differences in parity, calf birth weight, placental weight, or number of inseminations required to achieve pregnancy between IC and NIC (*P* > 0.05). Descriptive statistics and comparative results for these parameters are summarized in Table 3.

**Table 3:** Effect of major metritis pathogens on calf weight, placental weight, and number of inseminations

	Groups		
Parameter	NIC	IC	*P* <
Parity	3.38 ± 0.42	2.39 ± 0.28	0.0981
Number of Inseminations	2.13 ± 0.35	1.54 ± 0.25	0.2594
Calf weight	44.8 ± 3.2	43.5 ± 1.0	0.6328
Placenta weight	5.94 ± 0.43	6.47 ± 0.22	0.2709

IC, Infected Cows; NIC, Non Infected Cows

### Bacteriological Analysis Results

Bacteriological analysis revealed that 28 out of 36 cows (77.8%) were infected with at least one major metritis pathogen within the first 10 days postpartum. The remaining 8 cows (22.2%) constituted the NIC group. The most frequently isolated pathogen was *T. pyogenes* (50%), followed by *E. coli* (25%), *F. necrophorum* (14.2%), and *P. melaninogenica* (10.7%) in IC (Table 4).

**Table 4:** Isolation rates of major metritis pathogens

Growth status of major pathogens	Bacterial species *n* (%)	*n* (%)
**No growth**	-	8 (%22.2)
**Growth present**	*T. pyogenes*, 14 (%50)	28 (%77.8)
	*E. coli*, 7 (%25)	
	*F. necrophorum*, 4 (%14.2)	
	*P. meloninagenica*,* 3* (%10.7)	
**Total**		36 (%100)

### Serum and Placental Trace Element Concentrations at Parturition (D0)

At parturition, serum element profiles differed between IC and NIC (Table 5). Serum Mn concentrations were significantly lower in the IC group compared with the NIC group (0.43 ± 0.12 vs. 1.28 ± 0.62 mg/dL, *p* = 0.0348). Similarly, Fe concentrations were markedly reduced in the IC group (20.4 ± 3.2 vs. 57.2 ± 18.7 mg/dL, *p* = 0.0025). Serum Cu and Se levels were also lower in the IC group, whereas Zn concentrations were higher; however, the differences in Zn concentrations were not statistically significant (*p* > 0.05).

**Table 5:** Serum and placental trace element concentrations (mean ± SD) at parturition (D0) in NIC and IC groups

	Groups		
Serum (mg/dL)	NIC	IC	*P* <
Mn	1.28 ± 0.62	0.43 ± 0.12	0.0348
Fe	57.2 ± 18.7	20.4 ± 3.2	0.0025
Cu	5.04 ± 3.10	1.85 ± 0.49	0.0969
Zn	1.30 ± 0.25	1.66 ± 0.15	0.2484
Se	1.78 ± 0.73	0.80 ± 0.21	0.0830
**Placenta (mg/100 g)**			
Mn	0.63 ± 0.14	0.44 ± 0.12	0.4092
Fe	22.2 ± 6.9	20.1 ± 2.3	0.7099
Cu	1.50 ± 0.63	0.87 ± 0.16	0.1646
Zn	2.35 ± 0.41	3.19 ± 0.35	0.2318
Se	1.73 ± 0.58	0.98 ± 0.25	0.1869

Values are presented as mean ± SD. P-values were obtained using the general linear model (PROC GLM) to compare NIC and IC groups at parturition (D0). IC, Infected Cows; NIC, Non Infected Cows

No significant differences were detected in placental element contents between the groups. Although Mn, Fe, Cu, and Se concentrations were lower and Zn concentrations were higher in IC, which were insignificance (*p* > 0.05).

### Postpartum Serum Element Dynamics (D2–D10)

**Fig. 1:**
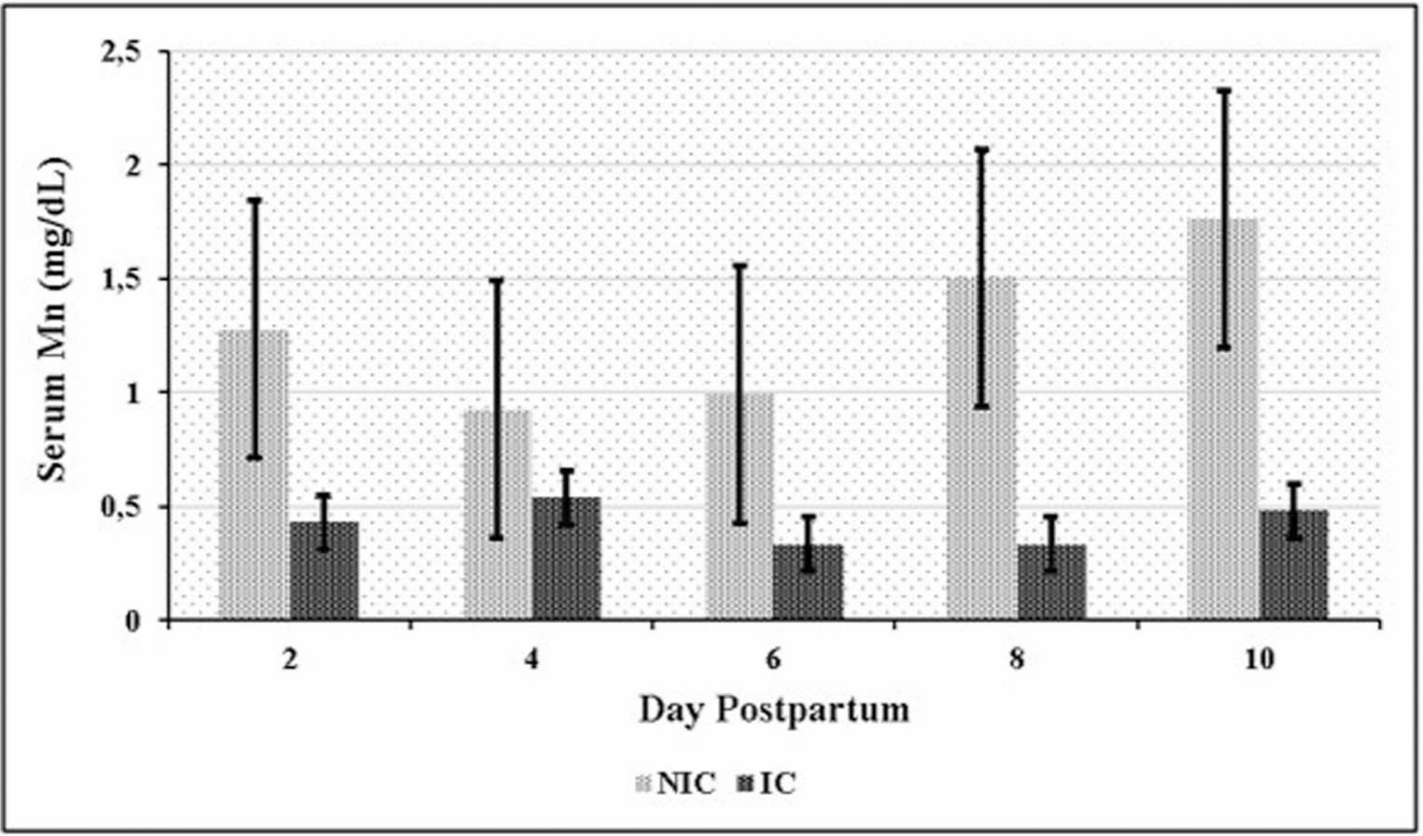
Serum manganese (Mn) levels in infected (IC) and non-infected cows (NIC)

**Fig. 2:**
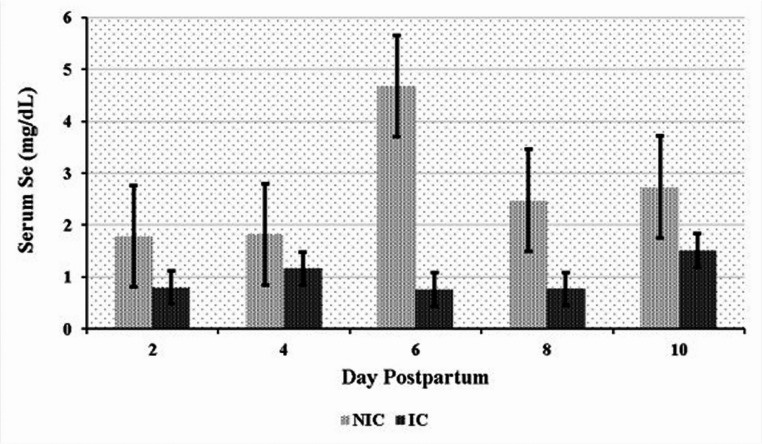
Serum selenium (Se) levels in infected (IC) and non-infected cows (NIC)

**Fig. 3:**
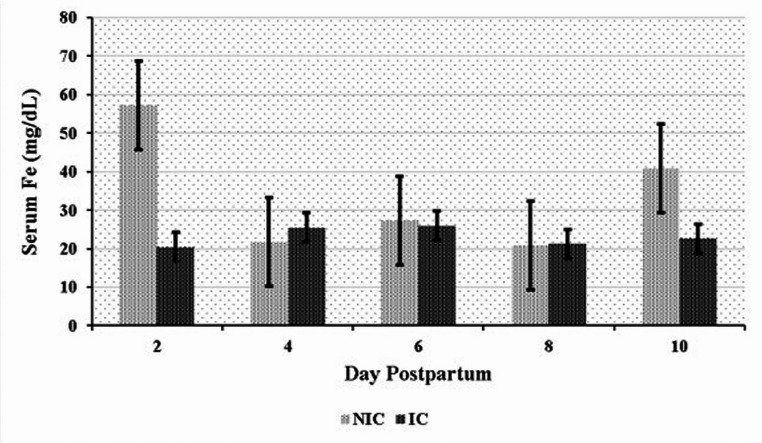
Serum iron (Fe) levels in infected (IC) and non-infected cows (NIC)

**Fig. 4:**
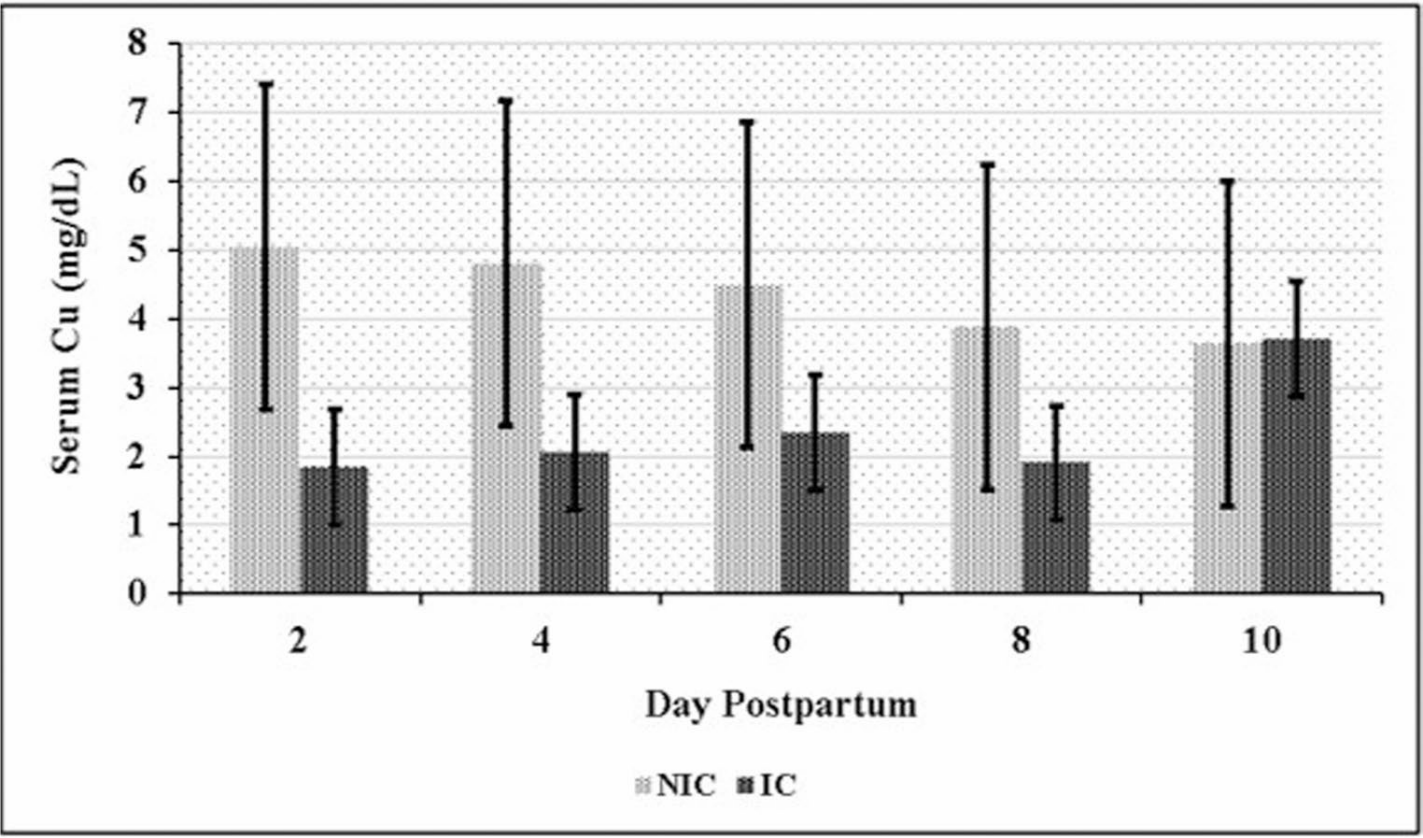
Serum copper (Cu) levels in infected (IC) and non-infected cows (NIC)

**Fig. 5:**
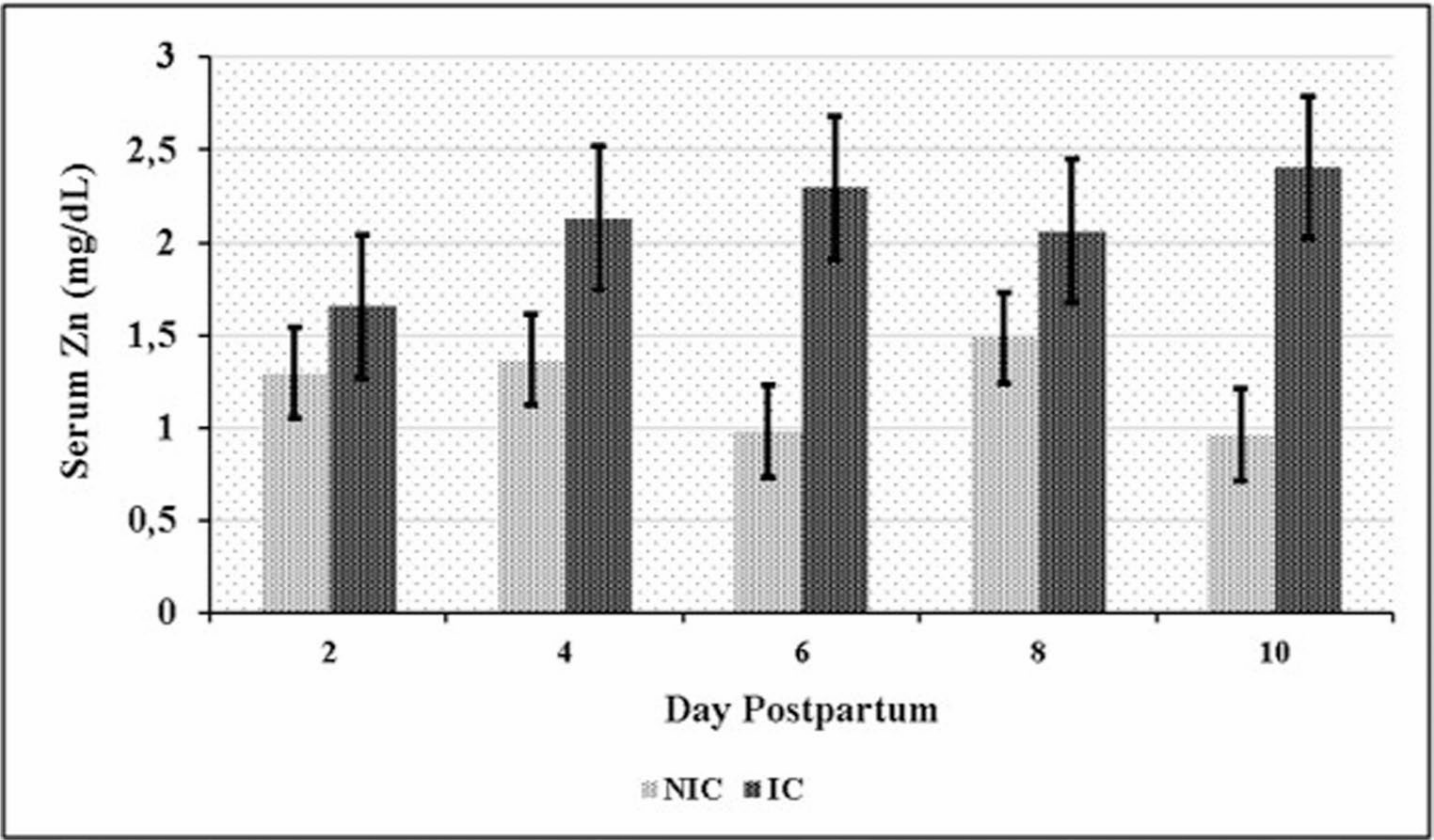
Serum zinc (Zn) levels in infected (IC) and non-infected cows (NIC)

Significant differences in serum element levels were observed between the NIC and IC groups during the postpartum period. NIC had significantly higher serum concentrations of Mn, Se, Fe, and Cu compared to IC (*P* < 0.05). Conversely, IC exhibited significantly higher serum Zn levels than NIC (*P* < 0.05) (Table 6). Time-dependent variations in element concentrations were also observed. In the NIC group, significant differences were noted for Mn on days 2, 8, and 10 (Fig. 1); for Se on days 6 and 8 (Fig. 2); for Fe on days 2 and 10 (Fig. 3); and for Cu on day 2 (Fig. 4) compared to the IC group. For Zn, significant differences were observed in the IC group on days 4, 6, and 10 (Fig. 5) (*P* < 0.05 or *P* < 0.01). On the other days, although not statistically significant, numerical trends were consistent with these findings. A significant Group x Time interaction was detected for Fe concentrations (*P* < 0.05), indicating divergent temporal patterns between the groups. A similar interaction trend was also observed for Se levels (Table 6).

**Table 6:** Postpartum (D2, 4, 6, 8, and 10) serum element concentrations in healthy cows and those with uterine infections

	Groups		*P* <		
Parameter	NIC	IC	Group	Time	Group x Time
Mn	1.28 ± 0.25	0.43 ± 0.05	0.0001	0.4639	0.4234
Fe	34.3 ± 6.5	23.1 ± 1.7	0.0256	0.1134	0.0256
Cu	4.41 ± 1.07	2.35 ± 0.39	0.0398	0.9919	0.8500
Zn	1.22 ± 0.11	2.10 ± 0.18	0.0139	0.9849	0.8457
Se	2.68 ± 0.49	0.99 ± 0.15	0.0001	0.1242	0.0576

Values are presented as mean ± SD. P-values for Group, Time, and Group x Time effects were obtained using repeated measures ANOVA. IC, Infected Cows; NIC, Non Infected Cows

### Correlation Analyses

Correlation analysis revealed a significant negative association between placental weight and serum Fe concentration (*r* = -0.43; *P* < 0.01). Additionally, significant positive correlations were found between serum and placental Cu concentrations (*r* = 0.47; *P* < 0.01) and between serum and placental Se concentrations (*r* = 0.32; *P* < 0.05) (Table 7). A positive correlation was also observed between parity and both calf birth weight (*r* = 0.54; *P* < 0.01) and placental weight (*r* = 0.29; *P* < 0.10) (Table 8).

**Table 7:** Pearson’s correlation coefficients among placental weight, and blood and placental element concentrations on the day of parturition (D0) (*r*)

	Pl-Wt	Pl-Mn	Pl-Fe	Pl-Cu	Pl-Zn	Pl-Se
**Pl-Wt**	1.00	0.05	-0.19	-0.09	-0.10	-0.15
**Sr-Mn**	0.07	0.01				
**Sr-Fe**	-0.43**		-0.06			
**Sr-Cu**	0.10			0.47**		
**Sr-Zn**	-0.16				0.02	
**Sr-Se**	-0.05					0.32*

*P < 0.05-*P* < 0.10, ** *P* < 0.01, Pl, Placenta; Sr, Serum; Wt, weight

**Table 8:** Pearson’s correlation coefficients among parity, calf weight, and placental weight (*r*)

	Parite	Number of Inseminations	Calf weight	Placenta weight
**Parite**	1.00	0.05	0.54^**^	0.29^*^
**Number of Inseminations**		1.00	0.01	-0.13
**Calf weight**			1,00	0.19
**Placenta weight**				1.00

^*^P < 0.05-*P* < 0.10, ^**^P < 0.01

## Discussion

In the present study, *Trueperella pyogenes* and *Escherichia coli* were identified as the most frequently isolated uterine pathogens in lochia samples collected during the early postpartum period. These observations fit within the broader understanding that postpartum uterine disease and transition-period inflammation are major determinants of subsequent reproductive performance in dairy cows [22, 23]. The predominance of these organisms is consistent with earlier reports [24–26], and their repeated isolation across consecutive sampling days underscores their ability to rapidly establish and persist within the postpartum uterus. Although clinical evaluations can reliably detect overt uterine infections, our findings reaffirm that subclinical colonization may also occur, highlighting the importance of microbiological monitoring during this critical period [9, 27]. The absence of these pathogens in some cows despite identical management conditions further suggests the presence of substantial individual variation in uterine immune competence, an observation previously linked to differences in oxidative and immunological responses [28, 29].

One of the key findings of this study was the significantly lower serum concentrations of Mn, Fe, Cu, and Se in infected cows. Considering the established roles of these trace elements in antioxidant defense, neutrophil function, and overall immune responsiveness [12, 14, 30, 31], these results indicate that inadequate mineral status may weaken postpartum uterine defenses and predispose cows to early uterine colonization. In contrast to these reductions, the higher serum Zn concentrations observed in infected cows are noteworthy and not entirely aligned with some previous reports [32]. However, both *T. pyogenes* and *E. coli* are known to activate potent inflammatory signaling pathways in endometrial epithelial and stromal cells [33, 34], suggesting that this elevation may reflect cytokine-driven inflammatory redistribution rather than true Zn sufficiency. Collectively, these findings support the notion that Zn may exhibit dynamic, time-dependent behavior during infection.

The potential influence of regional mineral availability in Erzurum should also be taken into account. Previous studies have shown natural variability in Cu, Mn, Fe, and Zn concentrations in local soils and forage sources, with some values falling below recommended levels for ruminants [35]. Nevertheless, because all cows in the present study received the same diet and water source, the considerable individual variation observed in serum mineral concentrations is likely attributable to physiological processes rather than environmental differences alone. This interpretation is consistent with evidence indicating that systemic inflammatory conditions alter circulating mineral distribution, increase mineral utilization, and negatively influence reproductive function [23, 36, 37]. Thus, the mineral discrepancies identified between infected and non-infected cows likely reflect a combined influence of both regional mineral supply and the metabolic and immunological dynamics inherent to the postpartum period.

Despite the pronounced differences in serum mineral concentrations, no significant differences were detected in placental mineral composition between the two groups. This finding indicates that placental mineral reserves do not necessarily mirror the systemic mineral status of the dam [38]. Nonetheless, given that placental tissue contributes directly to the composition of lochia -and that lochia provides the immediate microenvironment for bacterial proliferation [2, 9]- the possibility that placental mineral content indirectly shapes uterine microbial dynamics should not be dismissed. Future studies that simultaneously assess serum, placental, and lochia mineral profiles will be valuable for elucidating the mechanistic basis of these interactions.

The positive correlations observed among parity, placental weight, and calf birth weight are in line with previous reports in cattle and other species [39, 40]. The association between placental weight and calf birth weight reinforces the biological linkage between placental development and fetal growth [40, 41]. Likewise, the correlations detected between serum and placental Cu and Se concentrations support established maternal-fetal transfer mechanisms for these elements [42, 43]. Conversely, the negative correlation between serum Fe concentration and placental weight may indicate increased peripartum Fe mobilization toward placental tissues [42].

No significant differences were observed between infected and non-infected cows in the number of inseminations required to achieve conception. The uniformity in reproductive outcomes is likely attributable to consistent herd-level reproductive management, standardized nutritional support, and the capacity of some cows to spontaneously clear uterine pathogens during the early postpartum period [41–45]. Although statistical significance was not achieved, the numerical trends identified in our dataset suggest that larger cohorts or controlled experimental designs may be required to more clearly establish the extent to which mineral imbalance and uterine infection jointly influence reproductive performance.

Overall, the findings of this study demonstrate that postpartum trace mineral balance is closely associated with uterine microbial colonization and may influence both immune competence and the microscopic environment of the lochia. Future investigations incorporating simultaneous evaluation of serum, placental, and lochia mineral profiles will enhance our understanding of the pathogenesis of postpartum uterine infections and aid in the development of mineral-based strategies to improve reproductive outcomes in dairy herds.

### Limitations

This study has several limitations, and these aspects should be taken into consideration when interpreting the findings. First, the relatively small sample size and the fact that the research was conducted on a single farm, naturally limit the generalizability of the results to different management systems. In addition, the observational design of the study restricts the ability to establish a direct causal relationship between trace element concentrations and uterine infections. Although all cows on the farm consumed the same TMR and water source, it is well known that individual feed intake may not be completely uniform in free-stall housing systems. Variations in parity and individual metabolic status may also have contributed to the heterogeneity observed in trace element levels during this period. Furthermore, the absence of farm-specific soil, water, and feed element analyses represents a methodological limitation. Although biogeochemical data for the Erzurum region are available in the literature, the lack of an element profile specific to the farm where the research was conducted makes it difficult to fully characterize potential environmental influences. Finally, the physiological processes inherent to the postpartum period can naturally vary among individuals, and this variability may have contributed additional biological diversity to the interpretation of the trace element results.

## Conclusion

This study revealed associations between serum and placental trace element concentrations, the presence of major uterine pathogens, and certain reproductive parameters in Simmental cows during the early postpartum period. The findings suggest that trace element balance may influence not only immune responses but also the microscopic environment of the lochia. In particular, adequate levels of Mn, Se, Fe, and Cu may play an important role in maintaining uterine immune competence. As a practical approach for clinical monitoring of trace element status, periodic assessment of serum trace element concentrations during the close-up period may serve as an effective tool for the early detection of infections and the maintenance of uterine health. Furthermore, such integrative research could contribute to elucidating the mechanisms involved in the pathogenesis of uterine infections. Future studies with larger populations, extended follow-up periods, and interventional designs will help to clarify the observed associations and establish potential cause-effect.

## Data Availability

No datasets were generated or analysed during the current study.
